# Recurrence and profile of reconsultants: descriptive study of 162 patients

**DOI:** 10.1192/j.eurpsy.2023.646

**Published:** 2023-07-19

**Authors:** S. Brahim, W. BOUALI, K. Mohamed, K. Sabria, B. S. Rim, Y. Samira, Z. Lazhar

**Affiliations:** psychiatry, Taher Sfar, Mahdia, Tunisia

## Abstract

**Introduction:**

Emergency psychiatric consultation requests present certain specificities both in the situations encountered and in their management, due to a close relationship between the consultant and his environment. They do not only correspond to psychiatric emergencies, in the strict sense of the term, but also to psychological emergencies with their possible social dimension. They require an adapted response that can be decisive for the future

**Objectives:**

To determine the epidemiological and clinical characteristics of patients reconsultants in the emergency medical department.

**Methods:**

This is a cross-sectional study, conducted over a period of 12 months, from 01 April 2020 to 31 March 2021, in the emergency medical department of Mahdia University Hospital.

**Results:**

During the study period, 162 reconsultations for psychiatric emergencies were recorded. the age ranged from 18 to 61 years with an average of 35 years. The level of education was primary or secondary in 78.8% of cases. The majority of reconsultants had single marital status (70%). The absence of professional activity and social security coverage was found in 72.3 and 49% of cases respectively. The presence of family and personal psychiatric history was noted in 29.8% and 91.5% of reconsultants respectively. Heteroaggressiveness followed by instability were the most frequently encountered reasons for consultations with 23.4% and 12.8% of cases respectively. The presence of a triggering factor was found in 63.8% of cases where problems with the main support group followed by those related to the social environment and those related to access to health services were the most reported with 49.23 and 13% respectively. The syndromic psychiatric diagnoses were, in decreasing order of frequency, psychomotor excitement (23.4%), delusional syndrome (17%), dissociative syndrome (12.8%) and somatic conversion (12.8). For reconsultants, relational approach and/or injectable treatment were the most used therapeutic means immediatly (49%). Neuroleptics and benzodiazepines were prescribed in 38% and 13% of cases, respectively. The decision to hospitalize in a psychiatric department concerned 45% of reconsultants.

**Conclusions:**

Referral decisions favored the organization of ambulatory follow-up, with a decrease in the rate of hospitalization. These results make it possible to identify the evolutionary trends of the population consulting in emergency

**Disclosure of Interest:**

None Declared

